# Prevention and Communicability of Cholera

**Published:** 1866-02

**Authors:** 


### Editorial Department.

### Prevention and Communicability of Cholera.

Hygienists and Sanitarians appear to have multiplied or to have been supplied an appropriate field for labor. Laudable efforts are made to suggest what may be useful in preventing epidemic disease, and explain the causes and conditions which favor its appearance. The Citizens "Council of Hygiene and Public Health" of New York have published a report for the instruction of the public, giving a sketch of the progress and aspects of the approaching epidemic of cholera, drawing lessons from former epidemics and pointing out the natural fields and pestilential quarters in New York where cholera may be expected to prevail. They also show what may be the practical bearings of the teachings of the four former visitations of epidemic cholera in the United States, with what cholera will do, and what it will cost. Preventive measures are suggested, or what the people may do to prevent the ravages of an epidemic; particular duties are suggested with directions for cleansing and disinfection. This report has been published in pamphlet and widely distributed.

Buffalo Board of Health has not been inactive in this matter of sanitary regulations for the prevention of epidemic disease.—Health inspectors have been assigned to districts and all portions of the city are to be carefully inspected and nuisances abated. It would seem from a recent report of its action by Dr. Eastman, the Health Physician, that every reasonable effort is being made to place our city in the best condition known to sanitary science. Physicians held a meeting and a committee was appointed to act in concert with the Board of Health, if occasion should require, but it did not appear that anything of importance had been left this committee to suggest. The Board of Health is composed mostly of medical men, and the present intelligent Health Physician has been active in well directed efforts to increase the efficiency of the Board.

New York Council of Health have made a very full and able report, but so far as we are informed there has been as yet no *cleaning up.* New York is believed to be one of the dirtiest and consequently the most unhealthy city in the world; many of its inhabitants living in as great neglect of every hygienic law, as the Arabs, Greeks and Maltese, who were swept off in Alexandria at the first appearance of the present epidemic, which has been so fearful in Egypt.

In Buffalo we have not made very much demonstration, but we are told that we are being washed and disinfected, and we hope the work may be given to faithful hands, to men who will not take for proof that the sewer and vault are clean and free from obstruction, because the house stands upon Delaware street or Fifth Avenue, for we are satisfied that neglect in this matter is common even with the "oldest inhabitants."

We are daily asked in great earnestness, do you think we shall have cholera next year? and of course make reply in proportion to the time we have to devote to the question. We *shall* have cholera next year; we have made up our minds to have it, and Buffalo people have, what they make up their minds to have. We have had cholera, sporadic cholera, every year since its last appearance—had a disease which next summer we shall call cholera, and leave off all prefixes and qualifications. We hope not to have a fatal epidemic—not to die with cholera, for we have not made up our minds to die—are not ready to die—and Buffalo people have a pride in not doing what they have not made up their minds to do—are not ready to do.

It has been claimed for the Health Department of New York that they have "*Anchored Cholera in the Bay.*" More likely in their ditches, sewers, cesspools and cellars, and that warm weather will let it loose. Be that as it may, anchored, or not anchored, cholera springs up and propagates itself in foul places—in the crowded and filthy quarters of great cities; and we may also say in this connection, that it occurs separate and alone, without connection with others, and with no known sources of contagion. It is liable to become epidemic, and we know of no certain means of protection, nor are we acquainted with its modes of communication. The interesting letter of Prof. Lee, in this Journal, explains itself, and rather goes upon the presumption that the modes of communication in choleaa are now known, have been determined, have been entertained by the profession, and almost settled as fixed facts. It may have been suggested to some minds that the dejections in cholera were in some way capable of communicating the disease, in the same sense that the stools in typhoid fever have been believed to be capable of propagating that disease, but the profession in New York appear from Dr. Sayre's report to regard the suggestion that cholera is communicated by the dejections from the bowels and in no other way, as new, and though not settled, as yet worthy of consideration.

The suggestion is new, in the way it is presented in recent communications to this Journal, and we believe that nowhere can such a sentiment be found published in our literature of cholera. If it has been entertained, it has not been expressed in form to be understood, and the prevailing professional opinion concerning it has been, that it is due to atmospheric influences, localized perhaps, and rendered virulent by neglect of sanitary law.

This theory, which we believe to be essentially new, appears to us, as very likely to be also fallacious, certainly it is as yet unsustained by adequate proof. We have known cases of fatal cholera in men who have not left their own farms, or been visited by others having the disease. We have known many cases where the attendants suffered no ill effects; indeed it has been rare for hospital or private attendants upon cholera patients to suffer from the discharge of such duty. This was early observed, and soon led to a general belief in the non-contagion of cholera, a belief in contagion being unanimously entertained, both by the profession and public, at its first visitation. We hope never to have opportunity to test the truth of this suggestion, but if we do have such opportunity, may we go unbiased, and seek to know the truth only.

We publish a proposed plan for a quarantine station for cholera, by W. Marsden, M. D., etc., etc., of Quebec, which takes the ground that cholera is contagious, and proposes the most absolute isolation possible. The plan is an admirable one so far as securing that object is concerned, and if cholera is "*portable, communicable and controllable, and like plague to be transmitted and communicated both by persons and effects*," as stated in the preface to this plan, there cannot be too much said in favor of its adoption by the general government as a uniform system for the whole Union.

It must be apparent, however, to every one that these propositions as to the communicability of cholera are not wholly supported by fact and experience. Certainly we may safely say that its modes of communication are as yet undetermined, and more experiment and greater observation are required to settle this point, which has long been carefully considered, by the most accurate and intelligent observers. If these theories of communication should be sustained and generally adopted, the outbreak of cholera in onr cities would be attended by a panic as uncontrollable as that supposed to have occurred when Noah's ark was closed, and the windows of heaven opened—we should see re-enacted those awful scenes of horror witnessed at tne time when there was general belief in the contagion of the disease, husbands deserting their sick wives and children; wives fleeing from the dwellings which contained the dead bodies of their husbands; the sick unattended, and the dead unburied. Volumes have been already written to elucidate and explain these vexed questions, but God only knows what is true concerning them. If we attentively gather our facts, and carefully read the deductions which may be drawn from them, our understandings may yet be opened to know the truth.

